# Comparison of the antimicrobial efficacy of povidone-iodine-alcohol versus chlorhexidine-alcohol for surgical skin preparation on the aerobic and anaerobic skin flora of the shoulder region

**DOI:** 10.1186/s13756-020-00874-8

**Published:** 2021-01-22

**Authors:** Dorothea Dörfel, Matthias Maiwald, Georg Daeschlein, Gerald Müller, Robert Hudek, Ojan Assadian, Günter Kampf, Thomas Kohlmann, Julian Camill Harnoss, Axel Kramer

**Affiliations:** 1 Institute of Hygiene and Environmental Medicine, University Medicine Greifswald, Ferdinand-Sauerbruch-Strasse, 17475 Greifswald, Germany; 2 Department of Pathology and Laboratory Medicine, KK Women’s and Children’s Hospital, Singapore, Singapore; 3 Department of Microbiology and Immunology, Yong Loo Lin School of Medicine, National University of Singapore, Singapore, Singapore; 4 Duke-National University of Singapore Postgraduate School of Medicine, Singapore, Singapore; 5 Department of Dermatology, University Medicine Greifswald, Greifswald, Germany; 6 Working Group Clinical Antisepsis, German Society of Hospital Hygiene, Berlin, Germany; 7 Shoulder and Elbow Surgery, Rhön-Klinikum AG, Bad Neustadt/Saale, Germany; 8 Regional Hospital Wiener Neustadt, Vienna, Austria; 9 Institute for Skin Integrity and Infection Prevention, University of Huddersfield, Huddersfield, UK; 10 Institute of Community Medicine, University Medicine Greifswald, Greifswald, Germany; 11 Department of General, Visceral and Transplantation Surgery and Study Center, German Surgical Society, University Hospital Heidelberg, Heidelberg, Germany

**Keywords:** Skin antisepsis, Shoulder surgery, Chlorhexidine, Povidone-iodine, Alcohol

## Abstract

**Background:**

*Cutibacterium acnes* is part of the anaerobic skin microbiome and resides in deeper skin layers. The organism is an agent of surgical site infections (SSI) in shoulder surgery. We hypothesized that prolonged skin preparation with an agent that penetrates deeply into the skin would be beneficial. Thus, we compared two classes of antiseptics, each combined with alcohol, each applied with two different contact times.

**Methods:**

Using a cross-over arrangement, shoulders of 16 healthy volunteers were treated for 2.5 min (standard) or 30 min (prolonged) with alcohol-based chlorhexidine (CHG-ALC) or alcohol-based povidone-iodine (PVP-I-ALC). Skin sites were sampled before, immediately after, and 3 h after treatment, using a standardized cup-scrub technique.

**Results:**

Aerobic skin flora was reduced more effectively by PVP-I-ALC than by CHG-ALC after 2.5 min application and immediate sampling (reduction factor [RF] 2.55 ± 0.75 vs. 1.94 ± 0.91, p = 0.04), but not after prolonged contact times and 3-h sampling. Coagulase-negative staphylococci were completely eliminated after PVP-I-ALC application, but still recovered from 4 of 32 samples after CHG-ALC application. Anaerobic flora was reduced more effectively by PVP-I-ALC than CHG-ALC after standard (RF 3.96 ± 1.46 vs. 1.74 ± 1.24, p < 0.01) and prolonged (RF 3.14 ± 1.20 vs. 1.38 ± 1.16, p < 0.01) contact times and immediate sampling, but not after 3-h sampling. No adverse events were reported.

**Conclusions:**

PVP-I-ALC showed marginal benefits concerning the aerobic flora, but more substantial benefits over CHG-ALC concerning the anaerobic flora of the shoulder. Standard and prolonged contact times showed superiority for PVP-I-ALC for anaerobic flora at all immediate sampling points, but missed significance at 3-h sampling. The results underscore the need for protection against *C. acnes* and coagulase-negative staphylococci in orthopaedic surgery. The clinical relevance of these findings, however, should be studied with SSI as an endpoint.

## Introduction

The skin flora of patients is one of the most important factors in the pathogenesis of surgical site infections (SSI) [1–4]. Skin antisepsis constitutes an effective measure to reduce the numbers of microorganisms on skin. Therefore, it has been included as a key measure to prevent SSIs in recent international guidelines and recommendations [5–7].

The choice of the right preoperative skin antiseptic has been the topic of intense research, both microbiologically and in the form of clinical trials, and the subject of intense debate and controversies. The debate frequently focused on comparisons between “chlorhexidine and povidone-iodine” and which one of the two would be better; however, this did not take into account the important role of alcohols as potent ingredients in combination antiseptics [8]. In fact, many comparisons in the literature consisted of unequal two-against-one comparisons, for example, CHG-ALC combinations against aqueous PVP-I, or of comparisons of antiseptics with unknown or inadequate active ingredient content [9, 10]. Uncertainty surrounding these questions is also reflected by differences between recommendations in recent major guidelines; while the US Centers for Disease Control and Prevention guideline recommends an alcohol-based antiseptic with either CHG or PVP-I [6], the World Health Organization guideline recommends CHG-ALC over PVP-I-ALC [5].

The resident aerobic skin flora consists of organisms such as coagulase-negative staphylococci (CNS), *Micrococcus luteus, Corynebacterium* spp., *Malassezia furfur* and *Acinetobacter* spp. [11]. The anaerobic skin flora is located primarily in hair follicles and sebaceous glands. One of its main constituents is *Cutibacterium acnes* (formerly *Propionibacterium acnes*) [12, 13]. One study of lower limb surgery found common colonizing organism on skin and surgical wound edges to be CNS (80%), *Corynebacterium* spp. (25%) and *Cutibacterium* spp. (15%) [14]. Likely due to improvements in microbiological techniques, *C. acnes* is increasingly detected as a cause of SSIs, particularly in prosthetic joint infections [13].

In shoulder surgery, *C. acnes* predominate as the main anaerobic organism in SSI, particularly when prosthetic material is implanted [15–17]. The main reservoir for *C. acnes* is located deep in the skin within hair follicles and the pilo-sebaceous glands. In one study of superficial and deep intraoperative tissue samples collected during surgery, *C. acnes* was isolated in more than 36% of patients who received first-time shoulder surgery [15]. In another study, the chance of obtaining *C. acnes*-positive cultures was 2.5-fold greater in males and was smaller when patients reported to have hair loss [18]. In addition, *C. acnes* can be involved in infections after hip and knee joint replacements, after endo-prosthetic reconstructions of the femur [19], polyurethane-coated breast implants [20] and various other implants [21].

Thus, the anaerobic skin flora represents a major challenge for skin antisepsis. Lee et al. [22] reported that 7 out of 10 volunteers had *C. acnes* detectable in dermal punch biopsies after skin antisepsis with 2% chlorhexidine gluconate (CHG) with 70% isopropanol (IPA). Experiments with excised human skin in diffusion chambers showed that CHG penetrates relatively poorly into deep skin layers [23], while iodine released from povidone (PVP) molecules possesses substantially better penetration capabilities and penetrates through full-thickness skin in relevant concentrations in a time-dependent fashion [24]. Thus, PVP-I with alcohol (PVP-I-ALC) is hypothesised to have a greater antimicrobial effect against the deep resident skin flora when compared to CHG with alcohol (CHG-ALC). Although PVP-I in contrast to CHG has no appreciable residual antimicrobial effect [25], it exerts a long-lasting effectiveness on skin due to the delayed release of iodine from PVP-I by a second-order reaction.

The aim of this work was to conduct a study with healthy volunteers, following similar procedures as outlined by the US Food and Drug Administration (FDA) and the American Society for Testing and Materials (ASTM) [26–29], but modified to test the effects on both aerobic and anaerobic skin flora after standard and prolonged application times on the shoulder region. Competitor antiseptics were a commercially-available 2% w/v CHG with 55% w/v IPA preparation and a commercially-available antiseptic containing PVP-I and alcohol (3.24% w/v PVP-I, 38.9% w/v IPA, 37.3% w/v ethanol).

## Methods

### Study design

**Fig. 1:**
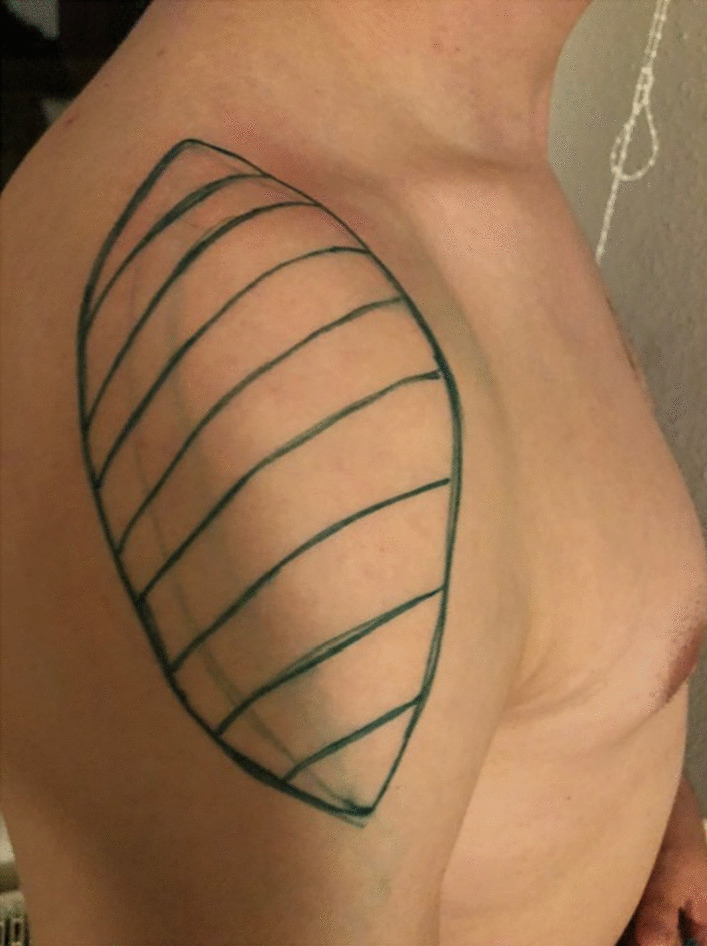
The sampled skin area is marked in green on a right male shoulder. Most of the incisional regions in arthroscopic and open shoulder surgery are included within this area

This study was conducted using a randomized cross-over design with participation of 16 healthy volunteers, 9 female and 7 male healthy individuals of Caucasian background and an average age of 31.3 years (range: 22–74 years). Two different skin antiseptics (CHG-ALC or PVP-I-ALC) and two different contact times for each antiseptic (2.5 min or 30 min) were applied on day 1 and 4, based on the assumption that a period of 3 days is sufficient for complete re-colonization of the skin. The application was carried out on both shoulders, using two separate shoulder areas for sampling (immediate and 3-h values) on the antero-lateral site of each shoulder on each day (Fig. 1). Participants were randomized by drawing opaque folded paper tickets from a container, such that two different antiseptic treatments per day were represented in a cross-over design, and each volunteer completed four different treatments. The Ethics Committee of the University of Greifswald approved the study (Reg. No. BB 109/10).

### Volunteers

Inclusion criteria were age > 18 years, legal capacity, informed consent after the study procedure was explained, as well as willingness and ability to comply with the test protocol. Exclusion criteria were macroscopically visible lesions of the skin except acne juvenilis, infections of the shoulder areas, dermatosis except acne, participation in another study within the last 30 days, pregnancy, lactation, thyroid disorders, intolerance to CHG or iodine, age under 18, therapy with radioactive iodine, antiseptic procedures on the designated areas within in the last 7 days and systemic antibiotics within the last 7 days. Two female volunteers suffered from active acne juvenilis at the shoulder areas that was less pronounced in the face; this was confirmed dermatologically.

### Tested compounds and application modes

Skin antisepsis was performed using the following commercially available products:

- ChloraPrep® (CHG 2% w/v, IPA 55% w/v [70% v/v], coloured; CareFusion, Leawood, USA) with applicators: vigorous rubbing using the 26 ml applicator for 30 s, afterwards keeping the treated area moist for 2 min with the antiseptic.
- Chlorhexidine 2% alcoholic (CHG 2% w/v, IPA 55% w/v [70% v/v]; B. Braun Medical, Sempach, CH): vigorous rubbing for 30 s using sterile forceps and gauze for 30 s, afterwards keeping the area moist with the antiseptic using a soaked sterile dressing (Zetuvit®, Hartmann, Heidenheim, Germany, 20 × 40 cm) for 29.5 min.
- Betaseptic® (3.24% w/v PVP-I, 38.9% w/v IPA, 37.3% w/v ethanol; Mundipharma, Limburg, Germany): vigorous rubbing for 30 s by using sterile forceps and gauze for 30 s, afterward keeping the area moist with the antiseptic by using forceps and gauze for 2 min or a soaked sterile dressing (Zetuvit®, 20 × 40 cm) for 29.5 min.

While the two CHG-ALC preparations had identical composition, one came with applicators, the other without. The preparation without applicators was necessary for the 30-min application, in order to keep the skin areas moist with soaked dressings for 30 min. For the two CHG-ALC products, the IPA percentage was converted to 55% w/v from the manufacturers’ stated 70% v/v for the purpose of uniformity of measurements.

### Sampling

Microbial skin counts were obtained before antiseptic application (pre-values), after application and air-drying of the sampling site (immediate post-values), and 3 h after treatment (3-h post-values). The cup-scrub technique according to ASTM E1874-14 [30] was used on 2.5 cm^2^ of skin, with 1 mL of sampling solution consisting of 0.9% sterile saline.

A first 10^–1^ dilution was prepared by adding 0.4 mL of sampling solution to 3.6 mL of neutralizer solution. For CHG-containing antiseptics, this was Lipofundin MCT 20% (B. Braun, Melsungen, Germany), for PVP-I-containing antiseptics, this was 3% Tween 80 (BioChemica, AppliChem, Darmstadt, Germany), 0.3% lecithin (AppliChem), 0.1% l-histidine (Roth, Nürnberg, Germany) and 0.5% sodium thiosulfate (Merck, Darmstadt, Germany).

Concurrently with the retrieval of the immediate post-values, another skin area of 4 × 4 cm was covered with a sterile dressing (Hydrofilm® transparent dressing 12 × 25 cm, Hartmann, Heidenheim, Germany) to protect a skin area where the 3-h samples were to be collected later.

### Microbiological techniques

After 5 min neutralization in the first 10^–1^ dilution, further dilutions of 10^–2^ and 10^–3^ were prepared in the respective neutralization solution, and 0.1 mL of each dilution was plated onto Columbia agar with 5% sheep blood (Becton Dickinson, Heidelberg, Germany) for aerobic incubation (37 °C, 48 h) and onto Schaedler agar (BioMérieux, Nürtingen, Germany) for anaerobic incubation (37 °C, 7 days). The anaerobic atmosphere was generated in anaerobic jars using Anaerocult A sachets (Merck, Darmstadt, Germany). After aerobic incubation, the colony forming units (CFU) were counted and a representative sample of colonies was picked for identification, such that at least one colony of each morphologically different colony type was tested. Isolates were subjected to simple phenotypic identification, including Gram stain, catalase and coagulase tests, followed by VITEK® Cards (BioMérieux). The anaerobic CFUs were counted after 7 days incubation, and again representative colonies were analysed by Gram stain and VITEK® Cards.

For uniformity of measurements, we converted the numbers of colonies counted to CFU per 5 cm^2^ of skin and expressed these as log_10_ values. Then, we calculated the reduction factors (RFs) as the differences between the log_10_ pre-values and the log_10_ post-values. To calculate the reduction factors and transform to log_10_, plates without growth were set to a value of 1.

### Sampling and validation of neutralization

Skin bacteria from five volunteers were collected by the cup-scrub technique [30] and pooled. Using the methodology of ASTM E1054-08 [31], pooled bacteria were used to verify the effectiveness of Lipofundin to inactivate CHG, of sodium thiosulfate to diminish the oxidizing agent iodine, and to ensure that the inactivation solutions did not significantly influence the bacterial counts, quantitatively and qualitatively. Final concentrations of 0.4% CHG and 0.6% PVP-I in 1 mL 0.9% NaCl were tested for neutralizer effectiveness. The concentration of the active agent was calculated using the treated skin area of 300 cm^2^ (17.5 cm × 17.5 cm) with 17 mL of antiseptic solution from the applicator and 3 mL of additional antiseptic solution, which arises from the equilibrium of the soaked dressing with the antiseptic liquid film on the skin. Finally, an area of 2.5 cm^2^ served as the basis for the microbiological examinations after scrubbing.

Validation of neutralization was conducted according to the methodology of ASTM E1054-08 [31]. The suitable neutralizers, lecithin for inactivating biguanides and thiosulfate for quenching iodine, were derived from Table 1 in ASTM E1054 [31] and Annex B in EN 1040 [32] and EN 13727 [33]. Lipofundin containing 1.2% egg yolk lecithin inactivated 0.4% CHG without any inhibitory effect on growth of pooled skin bacteria after aerobic and anaerobic incubation, and sodium thiosulfate, the quenching agent for iodine in the neutralization mixture, was effective for 0.6% PVP-I without influencing bacterial counts. Similar results were obtained in tests of neutralizer effectiveness, neutralizer toxicity and organism viability under aerobic and anaerobic culture conditions, using test solutions containing the residual antimicrobial agent which were derived from volunteers after skin antisepsis. Therefore, it was ascertained that CHG or PVP-I were effectively inactivated by the respective neutralization solutions without influencing bacterial growth after aerobic and anaerobic incubation.

**Table 1:** Efficacy of chlorhexidine-alcohol (CHG-ALC) versus povidone-iodine-alcohol (PVP-I-ALC) against aerobic and anaerobic flora at 2.5 and 30 min contact time; immediate and 3-h values

Preparation	Contact time (min)	Pre-values (log_10_)	Immediate effect		3-h effect	
			RF^a^	n (0 cfu)^b^	RF^a^	n (0 cfu)^b^
*Aerobic values*						
CHG-ALC	2.5	2.26 ± 0.93	1.94 ± 0.91	13	1.74 ± 1.08	10
	30	2.17 ± 0.73	2.17 ± 0.73	16	1.93 ± 0.92	13
PVP-I-ALC	2.5	2.55 ± 0.75	2.55 ± 0.75	16	2.25 ± 1.05	13
	30	2.20 ± 0.87	2.11 ± 0.93	15	1.94 ± 1.11	13
*Anaerobic values*						
CHG-ALC	2.5	3.99 ± 1.52	1.74 ± 1.24	6	1.46 ± 1.23	4
	30	3.55 ± 1.52	1.38 ± 1.16	7	1.59 ± 1.85	7
PVP-I-ALC	2.5	4.24 ± 1.27	3.96 ± 1.46	12	2.14 ± 1.65	4
	30	3.50 ± 1.40	3.14 ± 1.20	13	2.71 ± 1.36	10

^a^Mean reduction factor (RF) and standard deviation

^b^Number of volunteers with “0” cfu counts

### Statistical analysis

The analysis of the raw data was performed using Graphpad Prism (GraphPad, La Jolla, CA, USA) and SPSS (IBM, Armonk, NY, USA) software. Mann–Whitney and Wilcoxon tests were calculated. A p-value < 0.05 was considered statistically significant. Presence of carry-over effects was tested using linear mixed models, including treatment, sequence, period and treatment × period interaction effects (MIXED procedure in SPSS). Sample size calculations for testing differences in RFs were based on two-sided Wilcoxon signed-rank tests for matched pairs at p = 0.05. Effect sizes (i.e. differences in RFs divided by the standard deviation of the differences) of 0.75 and 1.0 were assumed. Based on a required power of 0.80, results of sample size calculation indicated that a sample between n = 10 and n = 16 cases was required (G*Power 3.1).

### Skin tolerability

All volunteers received a questionnaire for self-assessment of skin tolerability to evaluate the following parameters on an analogue scale from 1 to 10. Items “redness”, “burning”, “pruritus”, “scaliness”, and “pain” were assessed. In case of skin irritation, volunteers were asked to contact the investigators to have the nature of the irritation evaluated, and if necessary, to obtain treatment.

## Results

### Skin tolerability

The skin antiseptics were well tolerated after 2.5 and 30 min exposure without any irritations. None of the volunteers reported any of the five listed adverse events on the skin tolerability scale.

### Pre-values

The validity of the cross-over design was confirmed by a comparison of the pre-values on day 1 and day 4. There was no significant difference (mean log value day 1 aerobically, 2.23, standard deviation [SD], 0.80, 95% confidence interval [CI], 1.80–2.66; mean log value day 4 aerobically, 2.17, SD, 0.88, 95% CI, 1.70–2.64; mean log value day 1 anaerobically, 3.68, SD, 1.47, 95% CI, 2.90–4.46; mean log value day 4 anaerobically, 3.80, SD 1.44, 95% CI, 3.03–4.57).

### Aerobic skin flora

The aerobic flora consisted of more than 70% of CNS (mainly *S. epidermidis, S. hominis, S. saprophyticus* and *S. lugdunensis*). In addition, *S. aureus* (6% of aerobic flora) and *M. luteus* were found on the aerobic plates. PVP-I-ALC was significantly more effective than CHG-ALC when applied for 2.5 min, at the sampling time immediately after application (Tables 1 and 2), but this was not the case for the prolonged application time of 30 min and not for any 3-h values. There was no difference between the short and prolonged application times for each of the antiseptic agents, both immediately and 3 h after application (p = 0.09 and p = 0.16 for PVP-I-ALC; p = 0.37 and p = 0.51 for CHG-ALC). No statistically significant period or sequence effects or treatment × period interaction were found (p-values for immediate effect: 0.911 [period], 0.175 [sequence] and 0.987 [treatment × period]; p-values for 3 h effect: 0.197 [period], 0.213 [sequence] and 0.489 [treatment × period]).

**Table 2:** p values (Wilcoxon test) for the reduction of aerobic and anaerobic flora by chlorhexidine-alcohol (CHG-ALC) and povidone-iodine-alcohol (PVP-I-ALC) after 2.5 and 30 min contact time; immediate and 3-h values

Preparation	CHG-ALC 2.5 min contact		CHG-ALC 30 min contact	
	Immediate	After 3 h	Immediate	After 3 h
*Aerobic values*				
PVP-I-ALC 2.5 min contact	0.04	0.08	0.13	0.14
PVP-I-ALC 30 min contact	0.78	0.53	0.54^a^	0.95
*Anaerobic values*				
PVP-I-ALC 2.5 min contact	< 0.01	0.31	< 0.01	0.28
PVP-I-ALC 30 min contact	< 0.01	< 0.01	< 0.01	0.06^b^

^a^This comparison was numerically in favor of CHG-ALC; all other comparisons were numerically in favor of PVP-I-ALC

^b^When samples without detectable CFU were excluded from the analysis of 3-h post-values, the p value was 0.03

While CNS were not found in any samples after PVP-I-ALC treatment under any of the application and sampling conditions, they were still recovered with counts of up to 5 CFU per sample after CHG-ALC treatment from 3 of 16 samples after 2.5-min application and 1 of 16 samples after 30 min application and immediate sampling, respectively. None of the samples collected after 3 h yielded CNS.

### Anaerobic skin flora

The majority of the bacteria on anaerobic plates were *C. acnes*. Only a negligible number of other anaerobic species were recovered and a few anaerobic cultures yielded CNS. When comparing the RFs of the immediate post-values of PVP-I-ALC to the immediate post-values of CHG-ALC, the antisepsis with PVP-I-ALC was significantly more effective for both application times (Tables 1 and 2). Comparing the 3-h post-values, PVP-I-ALC applied for 30 min was significantly more effective than CHG-ALC applied for 2.5 min (p < 0.01), but not more effective than CHG-ALC applied for 30 min (p = 0.06; Table 2). When looking at the short (2.5 min) versus prolonged (30 min) application times, there was only one significant difference, in that PVP-I-ALC applied for 2.5 min appeared better than PVP-I-ALC applied for 30 min after immediate sampling, while all other 2.5–30 min comparisons showed no significant differences (p = 0.03 and p = 0.24 for PVP-I-ALC; p = 0.36 and p = 0.87 for CHG-ALC). However, we consider this single significant value a likely experimental outlier, because the log pre-values for the 2.5 min application of PVP-I were substantially higher (4.24 ± 1.27) than those for the 30 min application (3.50 ± 1.40), and in both instances, there was a majority of immediate post-values (12 and 13 of 16, respectively) with no CFU counts. Again, no statistically significant period or sequence effects or treatment × period interaction were found (p-values for immediate effect: 0.537 [period], 0.568 [sequence] and 0.584 [treatment × period]; p-values for 3 h effect: 0.392 [period], 0.230 [sequence] and 0.710 [treatment × period]).

## Discussion

The results of our study demonstrate that both antiseptic compounds, CHG-ALC and PVP-I-ALC effectively decreased the aerobic skin flora at shoulder sites. No growth at all was observed in 60 of 64 immediate post-values and in 49 of 64 3-h post-values. In aerobic cultures, PVP-I-ALC was better than CHG-ALC after 2.5 min contact time and immediate sampling (Table 2), but not in any other of the tested parameters, including prolonged application time and sampling after 3 h. The relative improvement of performance of CHG-ALC after prolonged application and late sampling appears consistent with the relatively slow skin penetration kinetics of CHG [23].

CHG-ALC did not completely eliminate CNS in some samples. This is consistent with findings from an earlier study in which CNS were frequently detected in the surgical field after 3 min of preoperative skin antisepsis with CHG-ALC (unpublished findings). This is also consistent with data from another study [4] that showed growth of residual bacteria directly after skin antisepsis with 70% v/v isopropanol in 35% of operations in orthopedic surgery. Among the isolates, 53% were identified as CNS [4]. These data underscore the need for protection against CNS in surgery by potent antisepsis.

More pronounced differences between the antiseptic compounds became apparent when tested against the anaerobic skin flora. PVP-I-ALC, when applied for 2.5 or 30 min, was better than CHG-ALC at all four immediate sampling points (Table 2), but not at the relevant 3-h sampling points when both received the equivalent applications times of 2.5 and 30 min, respectively. Overall, 6 of a total of 16 mean RFs obtained in this study were statistically significantly better for PVP-I-ALC than for CHG-ALC, and 15 out of 16 total measurement comparisons were in simple numerical terms better for PVP-I-ALC (Table 1). On the other hand, no relevant statistically significant differences were observed in any comparisons of the same agents between 2.5 min and 30 min application time.

Our results are in line with a clinical trial that compared the treatment of abdominal incision sites [34]. In this trial, 0.7% iodine povacrylex with 74% IPA was more effective in reducing SSI than 2% CHG with 70% IPA [34]. Another trial comparing 0.5% CHG with 70% alcohol and 1% PVP-I with 70% alcohol in hip and knee arthroplasty showed no difference in superficial wound complications [35]. However, on secondary endpoint analysis, skin antisepsis with CHG-ALC was associated with significantly higher odd ratios for overall SSI, including prosthetic joint infection [35]. In another trial, skin antisepsis with 7.5% PVP-I in aqueous solution did not show a significant difference in SSI rates to 2% CHG in 70% IPA after neurosurgical spine procedures in adults [36].

The shoulder region was chosen as test area because it is known to be colonized with *C. acnes* and has a high density of hair follicles and sebaceous glands [37]. *C. acnes* is a major pathogen of SSI in shoulder surgery [38, 39]. The standard method for preoperative skin antisepsis consists of treating the skin with either an applicator or with forceps and gauze for 30 s, followed by keeping the skin moist with the antiseptic for 2 min. The efficacy of both application methods is similar [40].

It is considered beneficial to use an extended contact time on skin areas that have a high density of sebaceous glands [41]. According to the manufacturer of the PVP-I-ALC solution that we used, the product’s recommended contact times are 1 min for skin with a low density and 10 min for skin with a high density of sebaceous glands. The contact time of 30 min in the present study was chosen because we reasoned that with an extended contact time, the deep skin flora would be targeted more effectively. If confirmed, this would have considerable implications for antiseptic preparation of the shoulder area. Indeed, after a contact time of 29.5 min under a soaked sterile dressing, the area still appeared to be moist.

It is thought that the physiological flora of the human skin is regenerated completely after 3 days, because the re-colonization starts about 60 min after alcohol-based skin antisepsis [42]. After 24 h, the skin flora is nearly completely regenerated [43]. Thus, the study was performed as a crossover study with an interval of 3 days between the tests. In line with our hypothesis, there was no significant difference between the pre-values on day 1 and day 4.

The validity and interpretation of our results depends heavily on the selection of effective neutralizing solutions. While this applies to all antiseptics, it is particularly relevant to CHG, because false-positive efficacy assessment in the absence of adequate neutralizers has been reported [44]. Soy bean or egg yolk lecithin is considered to be an effective neutralizer for CHG, and we used Lipofundin, which contains 1.2% egg yolk lecithin and has been previously shown to be suitable and effective [45].

Our study has two important limitations. First, an ideal trial should compare CHG and PVP-I combined with the same alcohol species with identical concentrations, if conclusions concerning the activity of the CHG or PVP-I component are to be made. However, we had to use readily available commercial formulations, due to the fact that PVP-I formulations are too difficult to prepare in-house. The CHG comparator contained 55% w/v (70% v/v) straight IPA, and the PVP-I comparator contained 76% w/v of a mix between IPA and ethanol in nearly equal parts. Therefore, there is a possibility that this study’s main results may be due to the different alcohol compositions. Future studies may be able to address the microbicidal activity of CHG and PVP-I when combined with equal comparator alcohols. Second, a majority of our immediate and 3-h post-values had no detectable CFUs, and this was more frequently observed for PVI-ALC than for CHG-ALC (Table 1). This means that our study unintentionally captured measurement values that were commonly located at the bottom end of the measurable range of the experimental setup. This may be owed to the experimental conditions, including the size of the sampled skin sites and sample fractions plated on agar media, in combination with the relatively small number of 16 participants. As a result, it appears likely that any differences between the two antiseptics, contact times and immediate versus 3-h effects were underestimated. A scenario in which a larger number of the measured values would be located well within the measurable range would have a greater chance of showing statistically significant differences if they exist. This appears particularly likely for the comparison of PVP-I-ALC versus CHG-ALC after 30 min contact time and sampling after 3 h, where the P value was 0.06, but 10 samples showed no detectable growth for PVP-I-ALC versus 7 for CHG-ALC. Future studies may be able to address this with larger numbers of volunteers, larger sampled skin sites, larger sample volumes or lower starting dilutions (e.g. neat, 10^–1^, 10^–2^ instead of 10^–1^, 10^–2^, 10^–3^) plated on agar, or a combination of these variables.

Commonly recommended contact times for surgical skin preparation, including the 2.5 min chosen in this study, are neither experimentally nor clinically well founded. Starting from the hypothesis that a prolonged application time achieves better penetration and reaches deeper skin compartments and hair follicles, we decided to examine a contact time of 30 min in addition to 2.5 min. However, no relevant significant differences were observed between these contact times. In addition, the question arises whether a contact time of 30 min is practicable in a busy operating room setting. This means that contact times shorter than 30 min should be investigated in future studies. For example, some antiseptic preparations, when applied for 2.5 min on areas with high density of sebaceous glands, meet or even exceed the efficacy of the experimental reference antiseptic that is applied for 10 min [46]. The 3-h values in our study aimed at assessing the sustained activity of the antiseptic and the intraoperative skin re-colonization under surgical drapes, as would be expected during typical operations.

Common efficacy testing of skin antisepsis only assesses aerobic flora, and in Europe the samples are typically collected by swabbing the skin surface [47] and do not mobilise the deep resident skin flora to the same extent as the ASTM cup scrub method does [30]. One possibility to address this in future studies might be to take dermal punch biopsies after antisepsis, so that the effects of prolonged contact times can be measured in deeper skin layers.

## Conclusions

While there was marginal superiority of PVP-I in combination with alcohol (3.24% w/v with ≥ 76% w/v alcohol) over 2% w/v CHG with 55% w/v IPA concerning the aerobic flora, there was more pronounced superiority concerning the anaerobic flora on the shoulder. PVP-I-ALC was clearly superior in its immediate efficacy in reducing the anaerobic skin flora. No significant difference was seen between standard and prolonged contact times of either agent. PVP-I-ALC seems to be a promising option for antisepsis on skin with a high density of sebaceous glands at a contact time of 2.5 min, especially in shoulder surgery. Future studies should expand upon these investigations with greater numbers of participants and contact times closer to 2.5 min, and ultimately should focus on clinical trials with SSIs as the endpoint.

## Data Availability

Original (de-identified) data are available from the corresponding author upon reasonable request.
